# Isolated but not combined ergogenic effects of caffeine and L-arginine during an isokinetic knee extension

**DOI:** 10.3389/fnut.2023.1303805

**Published:** 2024-01-08

**Authors:** Sebastian Zart, Maximilian Brachtendorf, Stephan Becker, Michael Fröhlich

**Affiliations:** Department of Sports Science, RPTU Kaiserslautern-Landau, Kaiserslautern, Germany

**Keywords:** ergogenic aids, synergistic effects, performance, muscle force, muscle endurance

## Abstract

**Introduction:**

The use of single supplements as ergogenic aids to enhance performance in strength-oriented sports is widespread among athletes (74%). The aim of this study was to increase knowledge about the combined effects of caffeine and L-arginine dietary supplements on performance.

**Methods:**

In this double-blind, randomized and counterbalanced crossover study, 29 participants (age: 23.2 ± 3.6 yr.; height: 181.0 ± 7.6 cm; weight: 77.0 ± 8.8 kg) each underwent six trials. In each trial performance tests were conducted to examine the effects of the supplement combinations on maximum (Nm_Max_) and averaged torque (Nm_M_), maximum (J_Max_) and averaged work (J_M_), the blockwise mean values of torque and work, and rate of perceived exertion (RPE) during an isokinetic leg extension task (90°·s^−1^) with the right leg for two sets of 40 repetitions and a set rest of 3 min on a dynamometer. The first and second trials were used to familiarize the participants with the movements in the dynamometer and no supplements were taken. After this 2-week pre-test trial, the supplement combinations of placebo/placebo, caffeine/placebo (5 mg·kg^−1^), L-arginine/placebo (0.15 g·kg^−1^), and caffeine/L-arginine (5 mg·kg^−1^ + 0.15 g·kg^−1^) were ingested.

**Results:**

The main finding of this study is the absence of an ergogenic effect of the combined supplements caffeine and L-arginine during voluntary maximal isokinetic leg extensions, although an increase of 3.5% was noted for Nm_max_ compared to the placebo trial. However, the administration of caffeine was able to increase the Nm_Max_ of the quadriceps femoris muscle about 5.1% (*p* = 0.043). In addition, caffeine (4.2%, *p* = 0.026) and also L-arginine (4.2%, *p* = 0.040) significantly increased Nm_M_ over a complete set. No single or combined supplement had an effect on muscle fatigue looking at the blockwise mean values of torque and work or RPE (all *p* > 0.05).

**Conclusion:**

The combination of caffeine and L-arginine was not superior to the isolated intake of both supplements in a strength-based exercise and a synergistic effect was absent.

## 1 Introduction

Approximately 74% of athletes report using caffeine as an ergogenic aid (1). The reason for this is likely due to the proven performance-enhancing effect in endurance and strength-oriented sports and tests. An umbrella review showed ergogenic effects for aerobic endurance (Cohen’s *d* = 0.22–0.61), muscle strength (*d* = 0.16–0.20), muscle endurance (*d* = 0.28–0.38), power (*d* = 0.18–0.27) and jumping performance (*d* = 0.17), among others (2). The amino acid L-arginine has also been studied to examine its effects on aerobic and anaerobic exercise (3). A meta-analysis on the effects of L-arginine supplementation reported more benefits for aerobic performance (standardized mean difference (SMD) = 0.84) than for anaerobic performance (SMD = 0.24), such as the Repeated Sprint Ability Test, strength exercises (isokinetic flexion/extension, bench press) or the Wingate Test (3). According to the meta-analysis, L-arginine is said to be effective at a dose of 0.15 g·kg^−1^. However, several systematic reviews made contradictory statements on the acute effects of L-arginine on strength-related parameters (4, 5).

Previous research has examined the effects of caffeine on maximum force development and fatigue resistance during repetitive strength exercise. For example, caffeine induced a significantly greater number of repetitions in participants during a strength endurance exercise (40% of 1 repetition maximum) when the performances in the caffeine conditions (3 and 6 mg·kg^−1^) were compared with the placebo condition of the first set (6). Similarly, in an isometric measurement of the leg extensors with a knee angle of 70°, it was found that caffeine supplementation (6 mg·kg^−1^) realized the maintenance of a given submaximal force for a longer time compared to the placebo condition (11.9%) (7). Additionally, in this study, participants increased maximum voluntary isometric strength by 5.9% (7). In another study, in which isokinetic strength of the leg extensors was measured at an angular velocity of 60°·s^−1^, significant differences were shown between the caffeine and placebo conditions for maximum torque (Hedges’ *g* = 0.30) and average power (Hedges’ *g* = 0.29) (8). The positive effect of caffeine on strength during isokinetic exercise at 60° and 180°·s^−1^ was also confirmed by a meta-analysis with a SMD value of 0.19 (95% CI = 0.06, 0.32, +6.1%) (9). A larger performance improvement of 7% for maximal voluntary contractions was evidenced by the meta-analysis of Warren et al. (10) with a SMD value of 0.37. Possible mechanisms explaining this increase in performance include improved activity of the sodium-potassium pump, calcium release in the muscle, and a delay in fatigue due to central nervous system effects (11).

Although the supplement L-arginine showed performance-enhancing effects in the meta-analysis listed above (3), it is worth mentioning in relation to the methodological approach of our study that the studies included in the meta-analysis did not demonstrate significant performance enhancement in isokinetic strength tests (12, 13). One explanation for the nonsignificant result of one study was an acute dose that was too low, representing only 50% of the amount of L-arginine described as effective (0.08 mg·kg^−1^) for the selected sample (13). This is also the problem with many other studies that investigated the acute effect of L-arginine on physiological parameters or performance increases and could not demonstrate an ergogenic effect (4). However, one study was able to measure an increase in nitric oxide production indirectly via NO^2−^, which is produced by the oxidation of NO by ceruloplasmin and through the binding of NO to the Cu^2+^ active site of cytochrome *c* oxidase and represents a good indication of endothelial nitric oxide synthase (eNOS) activity in humans. Since an increase in performance was also observed during high-intensity exercise, it could be assumed that L-arginine has ergogenic potential (14).

Based on the available literature, it is therefore assumed that the supplement caffeine has a performance-enhancing effect on strength parameters when taken in isolation and acutely (9). For L-arginine some studies only show performance-enhancing tendencies for strength parameters, whereby, with reference to Viribay et al. study, the dosage of L-arginine administered was too low. Specifically for isokinetic movements, there are no studies that show ergogenicity (3, 4). There are currently no studies on a combined effect of caffeine and L-arginine on muscle strength. Thus, the study aimed to investigate the isolated and combined effects of the two supplements caffeine and L-arginine without other concomitant substances on muscle strength during an isokinetic exercise.

## 2 Materials and methods

### 2.1 Sample

The sample consisted of 32 participants, which was reduced to 29 (age: 23.2 ± 3.6 yr.; height: 181.0 ± 7.6 cm; weight: 77.0 ± 8.8 kg) after the pretests because three participants dropped out for personal reasons. The remaining 29 participants, four of whom were female, completed all testing and were included in the analysis. Only individuals aged 18–40 years were included as participants who did not consume other supplements (e.g., creatine, nitrates or bicarbonate) and were fully able to exercise and to attend all tests. Participants with a habitual intake of caffeinated beverages (e.g., coffee, espresso) were also included in the study. Thirteen participants consumed caffeine through their diet and estimated the average amount to be 169.23 ± 111.87 mg·d^−1^ (Minimum: 50 mg·d^−1^, Maximum: 450 mg·d^−1^). Consequently, subjects who complained of tendomuscular or joint pain and were therefore unable to demonstrate unrestricted exercise capacity were excluded from the study. The subjects were informed on the study objectives and gave their written consent to participate in the study. The study was planned and performed on the basis of the Declaration of Helsinki (15) and was approved by the responsible Ethics Board (2020/55) of the RPTU Kaiserslautern-Landau.

The sample calculation was determined following the studies of Viribay et al. (3) and Grgic and Pickering (9). The calculation using G*Power 3.1.9.2 (alpha error = 0.05, power = 0.8 and effect size = 0.22) resulted in a sample size of 30.

### 2.2 Design

The double-blind randomized crossover study comprised six trials, which consisted of two pretests (Pre1, Pre2) without supplement intake and four trials (placebo, caffeine, L-arginine, caffeine + L-arginine) with supplement intake. After the pretests, which were intended to familiarize the participants with the load and the dynamometer, the participants were divided into three groups of equal strength based on the maximum torque achieved in order to ensure a balanced order of supplements. In addition, participants were instructed to keep a food diary each week for 48 h prior to testing, including testing day. In addition, they were instructed that the diet on the days prior to testing should be the same for each test. No caffeine or L-arginine was allowed 24 h before testing. The participants were also made aware that they should not engage in any sporting activity or heavy physical work for 24 h prior to testing. There was a one-week break between testing to ensure an adequate wash-out period, which served to completely metabolize the active compounds (16, 17). Care was taken to ensure that the participants were tested on the same day and at the same time each day.

### 2.3 Interventions

Study participants were administered one of the following supplement combinations during the four trials according to their sequence affiliation:

1. placebo-placebo (PLA)
2. placebo-caffeine (CAF)
3. placebo-arginine (ARG)
4. caffeine-arginine (CAFARG)

The amount of caffeine (Caffeine Pur, Powerstar Food, Homburg, Germany) was 5 mg·kg^−1^ body weight (9) and the amount of L-arginine (Arginine High End Cranberry, Powerstar Food, Homburg, Germany) was 0.15 g·kg^−1^ body weight (3). The placebo (Maltodextrin 100, Sponser, Wollerau, Germany) was always administered in the same amount. Caffeine and the caffeine placebo were filled into empty capsules (Extrakt Manufaktur, Rösrath, Germany) according to body weight. L-arginine and its placebo were mixed as a powder with 300 mL of water and consumed orally. Each supplement combination was taken 1 h before the start of testing.

### 2.4 Measurements

To record the isokinetic force of the right knee extensors, a concentric force measurement was performed on the dynamometer (IsoMed 2000, D&R Ferstl GmbH, Hemau, Germany) at an angular velocity of 90°·s^−1^. The torque in Newton meters (Nm) and the work in joules (J) during knee extension were used for the analysis. The flexion movement was performed without the force input and was disregarded for the study. From the measured parameters, it was then possible to subsequently filter the maximum torque (Nm_max_) and maximum work (J_max_) done of the best trial from both series. In addition, the average torque (Nm_M_) and average work (J_M_) over all repetitions of the best series could be calculated. As a subjective measure, the rate of perceived exertion (RPE) during the measurements was also recorded using the Borg scale (18) after completion of the two sets.

### 2.5 Procedure

The test days of the participants started with the intake of the supplement combination. After a one-hour waiting period, the participants performed a prescribed exercise program (e.g., high knees run, walking lunges and squats) for 5 min, which served to warm up the leg muscles. After a recovery period of 90 s, five counter movement jumps were performed to activate the leg extensor muscles. A pause of 10 s was observed between the jumps. Immediately thereafter, the participants sat down on the dynamometer with a hip angle of 100°. The participants were fixed at the shoulders, hips and thigh of the test leg. The rotational axis of the dynamometer was aligned with the joint space between the femur and tibia of each subject. Static gravity correction was performed according to the manufacturer’s instructions. The range of motion was 80° and was set to 10–90° of knee flexion (0° being full extension). The test administrators instructed the participants to extend their knee with maximum force during each repetition and to perform the return of the knee to the flexed position without the use of force. The participants performed two sets of 40 repetitions each. There was a rest of 3 min in between. Immediately after the last repetition, perception of exertion was assessed using the Borg scale. After completion of the isokinetic strength measurement, the participants were asked to estimate which supplement combination they had been given at the beginning of the test day. In addition, any side effects that occurred were to be reported in conclusion.

### 2.6 Analysis

Data from the isokinetic measurements (Nm, J) were entered into Microsoft Excel 2019 (Redmond, WA, United States) and maximum and mean values were determined before importing the data into IBM SPSS 28 (Armonk, NY, United States) and calculating the statistics. The descriptive statistics are presented by average values (M) and standard deviations (SD). To determine differences between trials, repeated-measures analyses of variance (ANOVA) with Tukey *post hoc* procedures were performed for the variables Nm_Max_, Nm_M_, J_Max_, J_M_, and RPE. An additional repeated measures ANOVA with between-subjects factor trial was calculated for the blockwise mean values of torque (Nm_1–10_, Nm_11–20_, Nm_21–30_, Nm_31–40_) and work (J_1–10_, J_11–20_, J_21–30_, J_31–40_). Also, repeated measures ANOVA was calculated for the macronutrients (carbohydrates, fats, proteins) based on the dietary protocols. Inferential statistics were calculated after verification of the preconditions of normality (Shapiro–Wilk test), variance homogeneity (Levene test), and Box test. In the case of a missing normal distribution, the repeated measures ANOVA was retained because it is generally robust to non-normally distributed data (19). In the absence of sphericity, a Greenhouse–Geisser correction was applied up to an epsilon of 0.75, and a Huynh field correction was applied above that. The significance level was set at *p* < 0.05.

## 3 Results

**Figure 1:**
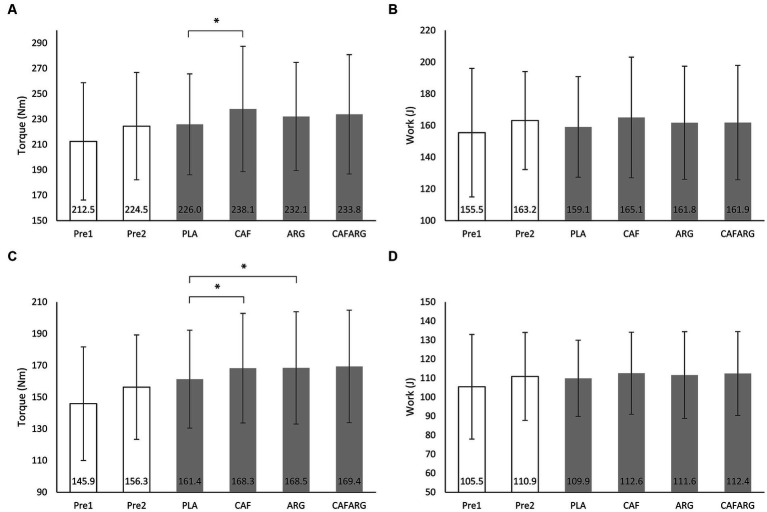
Maximum torque **(A)** and maximum work **(B)** from a series, average torque **(C)** and average work **(D)** across all repetitions of a series for the pretests (Pre1, Pre2) and the placebo (PLA), caffeine (CAF), L-arginine (ARG), and caffeine + L-arginine (CAFARG) trials. *p < 0.050.

In Table 1 and Figure 1 it can be seen that in the CAF, ARG, and CAFARG trials the variables Nm_Max_, Nm_M_, J_Max_, J_M_ always achieve greater values or percentage increases than in the PLA trial. Through the supplementation of caffeine, the majority of the highest values could be realized. An additional effect due to the combination of the two supplements cannot be identified from the descriptive analysis.

**Table 1:** Change in performance during an isokinetic leg extension in the CAF, ARG, and CAFARG trials compared to the PLA trial.

Variable	PLA	CAF	ARG	CAFARG	∆ PLA-CAF (%)	∆ PLA-ARG (%)	∆ PLA-CAFARG (%)
Nm_Max_	225.97 ± 38.81	238.07 ± 49.47	232.14 ± 42.67	233.83 ± 47.07	5.1*	2.9	3.5
Nm_M_	161.36 ± 30.90	168.28 ± 34.58	168.47 ± 35.42	169.37 ± 35.50	4.2*	4.2*	5.1
J_Max_	159.14 ± 31.72	165.10 ± 38.06	161.76 ± 35.93	161.86 ± 36.09	3.7	1.9	1.9
J_M_	109.91 ± 20.04	112.57 ± 21.58	111.63 ± 22.81	112.43 ± 22.05	2.5	1.4	2.4

ARG, L-arginine; CAF, caffeine; CAFARG, caffeine + L-arginine; J_M_, average work; J_Max_, maximum work; Nm_M_, average torque; Nm_Max_, maximum torque; PLA, placebo. *p < 0.05.

The variable Nm_Max_ showed a significant difference between the PLA and CAF trials, *F*(2.51, 70.32) = 3.616, *p* = 0.023, *η_p_*^2^ = 0.11 (*p* = 0.043; M_diff_ = −12.10, 95%-CI[−23.965, −0.242]). ANOVA also revealed a significant difference between trials for Nm_M_, *F*(3, 84) = 4.895, *p* = 0.003, *η_p_*^2^ = 0.15, distinguishing PLA and CAF (*p* = 0.026; M_diff_ = −6.920, 95%-CI[−13.252, −0.588]) and PLA and ARG (*p* = 0.040; M_diff_ = −7.110, 95%-CI[−13.998, −0.223]). Analyses on J_Max_ and J_M_ revealed no significant differences between trials (all *p* > 0.05).

**Figure 2:**
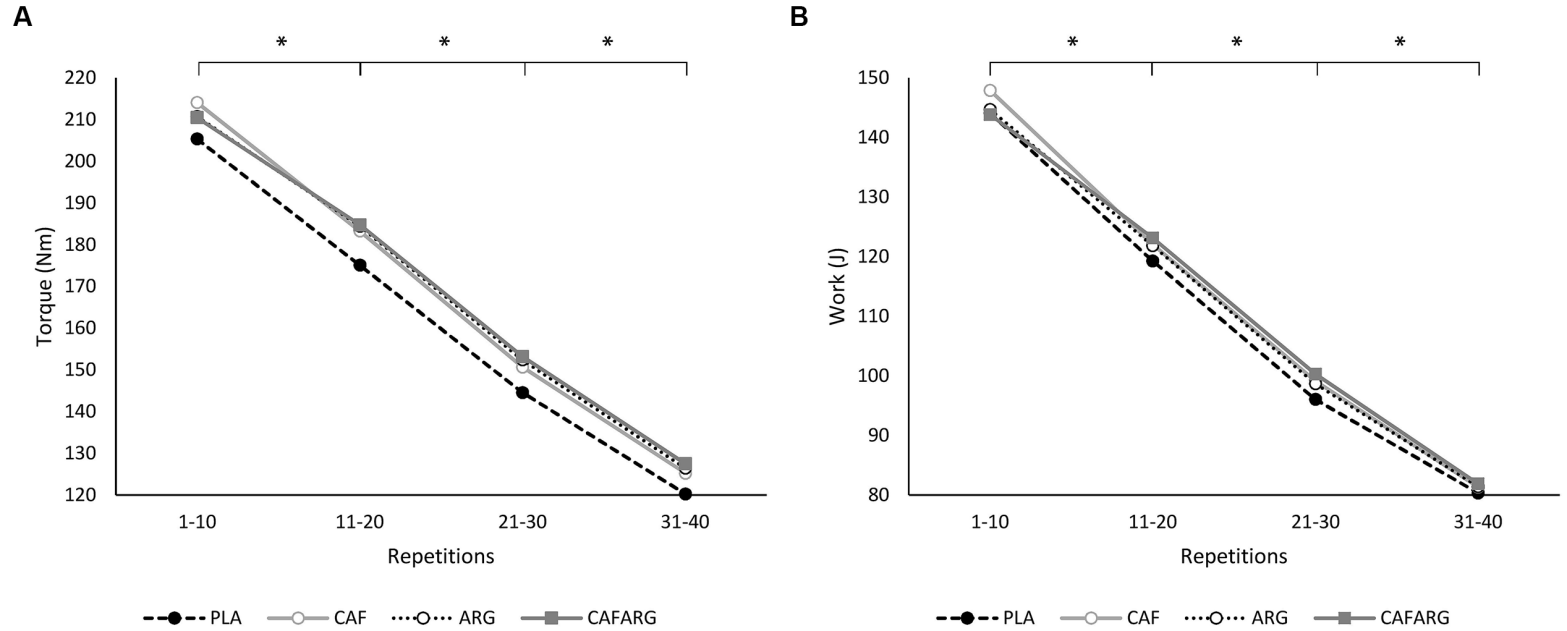
Group means of torque **(A)** and work **(B)** over the course of a series for the placebo (PLA), caffeine (CAF), L-arginine (ARG), and caffeine + L-arginine (CAFARG) trials. *p < 0.001.

Looking at the initial level and the power decrease within the sets of trials, it can be noted that the PLA trial started at a lower level, but the fatigue symptoms that occurred did not differ graphically between the trials (PLA, CAF, ARG, CAFARG) (Figure 2). This was more evident in the measured torques than in the work performed.

ANOVA revealed a significant main effect for the factor block with *F*(1.33, 148.57) = 738.593, *p* < 0.001, *η_p_*^2^ = 0.87 between blocks Nm_1–10_, Nm_11–20_, Nm_21–30_, Nm_31–40_ (all *p* < 0.001). However, no significant interaction effect occurred for block × trial, *F*(3.98, 148.57) = 0.727, *p* = 0.895, *η_p_*^2^ = 0.01. For the variable work, a significant reduction also occurred between blocks J_1–10_, J_11–20_, J_21–30_, J_31–40_ (all *p* < 0.001) with *F*(1.24, 138.99) = 512.101, *p* < 0.001, *η_p_*^2^ = 0.82. There was no interaction effect between block × trial with *F*(3.72, 138.99) = 0.231, *p* = 0.910, *η_p_*^2^ = 0.01.

Furthermore, no significant differences were found between trials with respect to RPE (all *p* > 0.05).

The query regarding the supplements administered showed that only 23.5% of the answers were correct. Thus, one of four trials in the participants’ answers corresponded to the actual supplement sequence. Only one participant correctly determined all trials.

In general, very few side effects were reported. Four participants reported slight nervousness in the ARG and CAF trials. One participant felt headache only during the CAFARG trial and two other participants complained of nausea (CAFARG), one of whom additionally reported nausea in the PLA and ARG trials.

With regard to dietary behavior, no significant differences were found for the macronutrients carbohydrates, fats and proteins between the trials (all *p* > 0.05).

## 4 Discussion

The primary result of this study and research question is a lack of ergogenic effect of the combination of the supplements caffeine and L-arginine during an isokinetic extension protocol of the right leg. However, it was found in the tests that the administration of caffeine was able to increase the maximum force development (Nm_Max_) of the quadriceps femoris muscle. In addition, caffeine and also L-arginine significantly increased the average torque (Nm_M_) over a set of 40 repetitions. An effect on RPE could not be achieved in any of the trials.

The results on CAF trial confirmed the positive effect by caffeine on increasing muscle strength and endurance (2, 9). Possible mechanisms are improvement of sodium-potassium pump activity, calcium release in muscle and delay of fatigue by central nervous system effects (11). For L-arginine, the effect on Nm_max_ in our study is not confirmed by the data, despite a slight increase. According to one study, there appear to be direct and indirect effects that influence contractile properties. Direct effects of NO lead to nitrosation or metal nitrosylation of target proteins. Among other things, this causes inhibition of muscle contraction through reduced force and shortening velocity. Indirect effects, on the other hand, are mediated by cyclic guanosine monophosphate (cGMP). The NO/cGMP system causes an immediate, reversible change in the contracting muscles by positively influencing the maximum force and the initial rate of force increase during a tetanic isometric contraction. Although this metabolic pathway can cause positive changes in muscle contraction, these effects are countered by the direct effects of NO, which bring about the opposite changes (brake action) and could partly cancel out the indirect effects of NO/cGMP (e.g., reduction of the initial rate of force increase) (20). However, arginine bioavailability and NO-dependent signaling was not measured in the current study and therefore we cannot evaluate these potential mechanisms of action.

Alvares et al. had three sets of 10 maximal isokinetic flexion movements of the elbow performed at 60°·s^−1^ and found a greater muscle blood volume during the set breaks, but an increase in maximal torque and absolute work did not occur (13). An opposite result is provided by the study of Campbell et al. (12), in which a significant increase in Nm_Max_ was reported. However, a comparison with this study is difficult because in Campbell et al., L-arginine α-ketoglutarate was not ingested as a single dose but at 12 g·d^−1^ for 3 weeks (12). Another study investigated the acute effect of the supplements caffeine (300 mg), L-arginine (3 g) and branched-chain amino acids (5 g) together with carbohydrates and a mixture of all supplements on repeated sprint ability (21). Although no supplement combination was able to significantly reduce the average and total sprint time, a statistical increase in peak power and average power was observed. Although the pairwise comparisons of the conditions were not significant, the participants with caffeine (peak power: 638.33 ± 147.12 W; average power: 564.17 ± 135.87 W) and L-arginine (peak power: 623.23 ± 129.96 W; average power: 542.45 ± 128.46 W) increased their power compared to carbohydrates (peak power: 596.11 ± 133.82 W; average power: 530.60 ± 138.47 W). With the intake of both supplements plus branched-chain amino acids and carbohydrates, the peak (655.97 ± 126.15 W) and average power (572.91 ± 115.23 W) could be increased even further. Despite the additional supplements contained, this is the first evidence of a synergistic effect of caffeine and L-arginine on performance (21).

In the current study, L-arginine was found to increase Nm_M_ over one set of isokinetic knee extensions. Despite methodological differences, this confirms the result of Stevens et al., who measured an increase in total work (J) during 35 isokinetic concentric-eccentric knee extensions after the intake of 6 g L-arginine plus 2 g glycine and 3.2 g α-ketoisocaproic acid (22). The improvement of Nm_M_ by L-arginine was shown in other studies by a higher, but only partially significant, endurance ratio or fatigue resistance index when set work or total work were used to determine the indices. These indices expressed a positive trend in fatigue rate between the placebo and L-arginine trials (13, 22). Our results could not confirm the lower drop in performance. When the repetition blocks were compared, fatigue occurred to the same extent regardless of the trial (Figure 2A). Only the level between the PLA trial and the supplements differed. To what extent an insufficient fatigue of the thigh muscles was provoked and a possible buffer capacity by L-arginine could possibly not be exhausted remains open. In a similar study, 100 isokinetic repetitions were performed with the leg extensor at 90°·s^−1^ and 20 repetitions each were averaged for comparison. The result also showed a continuous and significant decrease in torque between all blocks. Consequently, it is possible that our exercise protocol would have to include more repetitions or sets in order to demonstrate the possible effects of improved blood flow and thus increased buffering capacity. However, it is striking that from the beginning the torque of the ARG trial is more pronounced than in the PLA trial. Especially at the beginning of the set, this cannot have been caused by better blood flow or attributed to increased creatine synthesis by L-arginine (23). A more likely explanation could be the metabolic pathway of NO/cGMP that we mentioned above (20).

Although the combination of both supplements produced the greatest increase in Nm_M_ compared with the PLA trial, the differences from the CAF and ARG trials were small. The results clearly showed that there was no synergistic or additive effect from the combination of caffeine and L-arginine. Thus, there was no significant performance enhancement by the two supplements (21). One possible explanation could be an opposing physiological effect on the muscles by the two supplements. In an animal study, the induced caffeine caused an immediate reduction of all NOS isoforms in the muscle cells. Lower NOS expression led to lower NO production and increased contractile force. Administration of L-arginine partially reversed the effects of caffeine (24). Consequently, there could be a counterbalancing of the supplement effects.

In the end, it must be stated that the study design may have provoked too little fatigue at the local level in the stressed muscle to achieve the mentioned effects of the supplements. Despite positive effects with acute supplementation of L-arginine, a multi-day supply could be beneficial to achieve the desired physiological adaptations (3). The measurement of isokinetic movements with a dynamometer represents a special load for the muscles, therefore the transfer of the results to sport-specific and natural movements is limited. Furthermore, no measurements were taken of the amount of L-arginine in the blood and NO. The strengths of the study are the crossover design used, which allows an intra-individual comparison between the trials. In addition, the supplements were administered in isolated form without accompanying substances, so that effects could be determined directly from the measurements. In addition, the use of a dynamometer and the laboratory conditions enabled standardized test conditions.

## 5 Conclusion

When maximum torques were required several times during a knee extension series over a period of about 2 min, supplementation with caffeine was able to increase both maximum and average torque. The supplement L-arginine also achieved an increase in average torque. In contrast, the combination of both supplements did not result in a greater increase in performance.

## Data availability statement

The raw data supporting the conclusions of this article will be made available by the authors, without undue reservation.

## Ethics statement

The studies involving humans were approved by the Ethics Committee of the RPTU Kaiserslautern-Landau. The studies were conducted in accordance with the local legislation and institutional requirements. The participants provided their written informed consent to participate in this study.

## Author contributions

SZ: Conceptualization, Data curation, Formal analysis, Methodology, Visualization, Writing – original draft, Writing – review & editing. MB: Data curation, Investigation, Resources, Writing – original draft, Writing – review & editing. SB: Conceptualization, Methodology, Writing – review & editing. MF: Conceptualization, Methodology, Writing – review & editing.
